# Native High-Density Lipoproteins (HDL) with Higher Paraoxonase Exerts a Potent Antiviral Effect against SARS-CoV-2 (COVID-19), While Glycated HDL Lost the Antiviral Activity

**DOI:** 10.3390/antiox10020209

**Published:** 2021-02-01

**Authors:** Kyung-Hyun Cho, Jae-Ryong Kim, In-Chul Lee, Hyung-Jun Kwon

**Affiliations:** 1 Medical Innovation Complex, Korea Research Institute of Lipoproteins, Daegu 41061, Korea; 2 LipoLab, Yeungnam University, Gyeongsan 712-749, Korea; 3 Department of Biochemistry and Molecular Biology, Smart-Aging Convergence Research Center, College of Medicine, Yeungnam University, Daegu 705-717, Korea; kimjr000@gmail.com; 4 Functional Biomaterials Research Center, Korea Research Institute of Bioscience and Biotechnology, Jeongeup 56212, Korea; leeic@kribb.re.kr (I.-C.L.); hjkwon@kribb.re.kr (H.-J.K.)

**Keywords:** COVID-19, SARS-CoV-2, high-density lipoproteins (HDL), glycation, paraoxonase, low-density lipoproteins

## Abstract

Human high-density lipoproteins (HDL) show a broad spectrum of antiviral activity in terms of anti-infection. Although many reports have pointed out a correlation between a lower serum HDL-C and a higher risk of COVID-19 infection and progression, the in vitro antiviral activity of HDL against SARS-CoV-2 has not been reported. HDL functionality, such as antioxidant and anti-infection, can be impaired by oxidation and glycation and a change to pro-inflammatory properties. This study compared the antiviral activity of native HDL with glycated HDL via fructosylation and native low-density lipoproteins (LDL). After 72 h of fructosylation, glycated HDL showed a typical multimerized protein pattern with an elevation of yellowish fluorescence. Glycated HDL showed a smaller particle size with an ambiguous shape and a loss of paraoxonase activity up to 51% compared to native HDL. The phagocytosis of acetylated LDL was accelerated 1.3-fold by glycated HDL than native HDL. Native HDL showed 1.7 times higher cell viability and 3.6 times higher cytopathic effect (CPE) inhibition activity against SARS-CoV-2 than that of glycated HDL under 60 μg/mL (approximately final 2.2 μM) in a Vero E6 cell. Native HDL showed EC_50_ = 52.1 ± 1.1 μg/mL (approximately final 1.8 μM) for the CPE and CC_50_ = 79.4 ± 1.5 μg/mL (around 2.8 μM). The selective index (SI) of native HDL was calculated to be 1.52. In conclusion, native HDL shows potent antiviral activity against SARS-CoV-2 without cytotoxicity, while the glycation of HDL impairs its antiviral activity. These results may explain why patients with diabetes mellitus or hypertension are more sensitive to a COVID-19 infection and have a higher risk of mortality.

## 1. Introduction

High-density lipoproteins (HDL) are macromolecules that consist mainly of apolipoprotein (apo) A-I, cholesterol, and phospholipid in human serum. They are a key player in reverse cholesterol transport [1]. Native HDL exerts potent antioxidant and anti-inflammatory activity [2] with a broad spectrum of antiviral activity against various viruses, such as vaccinia, poliovirus, and herpes simplex virus [3]. Changes in plasma HDL-C have been reported to occur during viral infections, such as human immunodeficiency virus (HIV) [4], human hepatitis-C virus (HCV) [5], and hemorrhagic fever renal syndrome (HFRS) [6]. During a Hantaan viral infection, serum HDL-C was lowered remarkably in the oliguric phase with severe acute inflammation [7]. 

Coronavirus disease 2019 (COVID-19) is an ongoing pandemic caused by the severe acute respiratory syndrome coronavirus 2 (SARS-CoV-2), which was first identified in December 2019 in Wuhan, China. In March 2020, the World Health Organization (WHO) declared the outbreak of a pandemic. The global economy was then impacted adversely by lockdowns due to COVID-19, with an explosive increase in the number of patients. 

Patients with COVID-19 in Wenzhou, China, showed a remarkable decrease in HDL-C and an elevation of the monocyte/HDL-C ratio, particularly in subjects with a primary infection and male patients [8]. Female patients with a primary infection tended to recover at nine days post-infection and were discharged from the hospital after an elevation of HDL-C was noted [8]. Furthermore, the degree of hypolipidemia positively correlated with the COVID-19 severity [9]. In particular, the HDL-C level decreased according to the severity of the cytokine storm [10]. HDL-C is an important factor affecting virus clearance in COVID-19 patients [11]. A lower serum HDL-C level was significantly associated with the longer clearance of SARS-CoV-2, approximately 32.5 days after the onset of COVID-19 [11]. Other studies showed that the average time from the beginning of symptom onset to the first negative test of a throat swab SARS-CoV-2 was 9.5–11 days [12]. These results strongly suggest that high HDL-C levels might be beneficial in symptomatic patients with COVID-19 via the putative antiviral activity.

On the other hand, the beneficial effects of HDL can be impaired by undesirable modifications, such as oxidation and glycation, to produce dysfunctional HDL, which has more pro-atherogenic and pro-inflammatory properties. Patients with myocardial infarction [13] and angina pectoris [14] showed lowered HDL-C and dysfunctional HDL with modifications. HDL from the elderly showed impaired paraoxonase activity with truncated and multimerized apoA-I [15] via glycation [16] than those of the young control. Furthermore, young smokers also showed similar characteristics of a smaller HDL particle size with more oxidation and glycation [17], such as the characteristics of the elderly. The lethality of the SARS-CoV-2 is notably selective for the elderly, and those with chronic diseases, such as hypertension, diabetes, cardiovascular disease, and smoking [18]. HIV-1 infected patients also showed dysfunctional HDL with the progression of atherosclerosis [19]. Glycated apoA-I and reconstituted HDL showed severe structural and functional modification to accelerate atherosclerosis and senescence [20,21].

Overall, these reports suggest that HDL functionality, particularly paraoxonase (PON-1) activity, might be important for suppressing a SARS-CoV-2 infection. Native HDL and dysfunctional HDL might have different antiviral activities. This study tested and compared the antiviral activity of native HDL and glycated HDL against replication of SARS-CoV-2. 

## 2. Materials and Methods 

### 2.1. Purification of Lipoproteins

Low-density lipoprotein (LDL, 1.019 < *d* < 1.063) and high-density lipoprotein (HDL, 1.063 < *d* < 1.225) were isolated from young and healthy human plasma (Blood Bank of Yeungnam University Medical Center, Daegu, Korea) by sequential ultracentrifugation [15], and the density was adjusted by adding NaCl and NaBr in 10 mM Tris-HCl/140 mM NaCl (pH 7.4) using the standard protocols [22]. The samples were centrifuged for 22 h at 10 °C and 100,000× *g* using a Himac CP-100WX (Hitachi, Tokyo, Japan) at the Instrumental Analysis Center of Yeungnam University.

### 2.2. Glycation of HDL

The purified HDL (2 mg/mL) was incubated with 250 mM D-fructose in 200 mM potassium phosphate/0.02% sodium azide buffer (pH 7.4) for up to 72 h in air containing 5% CO_2_ at 37 °C. It has been known that fructose can induce remarkably higher glycation extent of apoA-I than glucose treatment in according to previous report [21]. The extent of glycation was determined by reading the fluorometric intensity at 370 nm (excitation) and 440 nm (emission), as described previously [23] using an LS55 spectrofluorometer (PerkinElmer, Shelton, CT, USA) and a 1 cm path-length suprasil quartz cuvette (Fisher Scientific, Pittsburg, PA, USA). 

### 2.3. Electron Microscopy

Transmission electron microscopy (TEM, Hitachi H-7600; Ibaraki, Japan) was performed at an acceleration voltage of 80 kV. HDL was negatively stained with 1% sodium phosphotungstate (PTA; pH 7.4) with a final apolipoprotein concentration of 0.3 mg/mL in TBS. Five μL of the HDL suspension was blotted with filter paper and replaced immediately with a 5 μL droplet of 1% PTA. After a few seconds, the stained HDL fraction was blotted onto a Formvar carbon-coated 300 mesh copper grid and then air-dried. The shape and size of HDL were determined by TEM at a magnification of 40,000× according to a previous report [6,7]. 

### 2.4. Paraoxonase Assay 

The paraoxonase-1 (PON-1) activity toward paraoxon was determined by evaluating the hydrolysis of paraoxon to *p*-nitrophenol and diethyl phosphate, which was catalyzed by the enzyme [24]. Equally diluted HDL (20 μL, 2 mg/mL) was added to 230 μL of a paraoxon-ethyl (Sigma Cat. No. D-9286) containing solution (90 mM Tris-HCl/3.6 mM NaCl/2 mM CaCl_2_ [pH 8.5]). The PON-1 activity was then determined by measuring the initial velocity of *p*-nitrophenol production at 37 °C, as determined by measuring the absorbance at 415 nm (microplate reader, Bio-Rad model 680; Bio-Rad, Hercules, CA, USA). 

### 2.5. Cell Culture

THP-1, a human monocyte cell line, was obtained from the American Type Culture Collection (ATCC, #TIB-202™; Manassas, VA, USA) and maintained in RPMI-1640 medium (Hyclone, Logan, UT, USA) supplemented with 10% fetal bovine serum (FBS) until needed. The cells that had undergone no more than 20 passages were incubated in a medium containing phorbol 12-myristate 13-acetate (PMA; final concentration, 150 nM) in 24-well plates for 48 h at 37 °C in a humidified incubator (5% CO_2_ and 95% air) to induce differentiation into macrophages. 

### 2.6. Acetylation of LDL and Phagocytosis Assay

The acetylation of LDL (acLDL) was performed using saturated sodium acetate and acetic anhydride according to the method described elsewhere [25]. After acetylation and subsequent dialysis, the acLDL protein content was determined and filtered through a 0.22 μm filter (Millex; Millipore, Bedford, MA, USA).

To compare the anti-atherosclerotic activity of each HDL, inhibition against phagocytosis of acLDL into macrophages was important to prevent foam cell formation, as reported elsewhere [26]. The differentiated and adherent macrophages were incubated with 400 μL of fresh RPMI-1640 medium containing 1% FBS, 50 μL of acLDL (1 mg of protein/mL in PBS), and 50 μL of each HDL (final concentration, 0.5 mg/mL of apoA-I) for 48 h at 37 °C in a humidified incubator. After incubation, the cells were washed three times with PBS, then fixed in 4% paraformaldehyde for 10 min. The fixed cells were rinsed with 100% polypropylene glycol, stained with an oil-red O staining solution (0.67%), and washed with distilled water. The THP-1 macrophage-derived foam cells were then observed and photographed using a Nikon Eclipse TE2000 microscope (Tokyo, Japan) at 600× magnification. Quantification of the oil-red O-stained areas in the cell was carried out via computer-assisted morphometry using Image Proplus software (version 4.5.1.22; Media Cybernetics, Bethesda, MD, USA). The cell media (0.2 mL) were then analyzed using a thiobarbituric acid reactive substance (TBARS) assay to evaluate the changes in levels of oxidized species using a malondialdehyde (MDA) standard.

### 2.7. SARS-CoV-2 and Cell Lines

In according to previous report [27], Vero E6 cell was employed to study cell viability and antiviral assay. Vero E6 (African green monkey cell line) cells were kindly provided by the American Type Culture Collection (ATCC CRL-1586; Manassas, VA, USA), and the SARS-CoV-2 strain (BetaCoV/Korea/KCDC03/2020: NCCP 43326) was obtained from National Culture Collection for Pathogens in Korea. Vero E6 cells were maintained in Dulbecco’s modified Eagle’s minimum (DMEM) supplemented with 5% fetal bovine serum (FBS), 100 U/mL penicillin, 100 μg/mL streptomycin, and 100 U/mL amphotericin B. The SARS-CoV-2 strain was propagated onto confluent Vero cells in the presence of 1 μg/mL tosyl phenylalanyl chloromethyl ketone (TPCK) trypsin, which was purchased from Sigma-Aldrich (St. Louis, MO, USA). 

### 2.8. Cytotoxicity

The Vero E6 cells were grown in 96 well plates at 1 × 10^5^ cells/well for 48 h. The cells were replaced with media containing serially diluted lipoprotein for 72 h. The solution was replaced with only media, and 10 μL of an MTT (3-(4,5-dimethylthiozol-2-yl)-3,5-diphenyl tetrazolium bromide, Sigma, St. Louis, MO) solution was added to each well and incubated at 37 °C for 4 h. After removing the supernatant, 100 μL of a 0.04 M HCl–isopropanol solution was added to dissolve the formazan crystals. The absorbance was measured at 540 nm with a subtraction of the background measurement at 655 nm in a microplate reader (Bio-Rad model 680). The 50% cytotoxic concentration (CC_50_) was calculated by regression analysis, as described previously [28].

### 2.9. CPE Reduction Assay

The SARS-CoV-2 strain at a 0.001 multiplicity of infection (MOI) was inoculated onto near confluent Vero E6 cell monolayers (1 × 10^5^ cells/well) for 1 h with occasional rocking. The medium was removed and replaced by DMEM with each lipoprotein at different concentrations. The cultures were incubated for 72 h at 37 °C under a 5% CO_2_ atmosphere until the cells in the infected, untreated control well showed a complete viral cytopathic effect (CPE), as observed by optical microscopy. Each lipoprotein was assayed for virus inhibition in triplicate.

After 72 h incubation in all antiviral assays, the cells were replaced with only media, and a 10 μL MTT solution was added to each well and incubated at 37 °C for 4 h. After removing the supernatant, 100 μL of a 0.04 M HCl–isopropanol solution was added to dissolve the formazan crystals. The absorbance was measured at 540 nm with a subtraction of the background measurement at 655 nm using a microplate reader (Bio-Rad model 680). The 50% inhibitory concentration (IC_50_) was calculated by regression analysis. The selective index (SI) was calculated using the formula, SI = CC_50_/IC_50_, as described previously [29].

### 2.10. Data Analysis

All data are expressed as the mean ± SD from at least three independent experiments with duplicate samples. A *p*-value <0.05 was considered significant. Statistical analysis was performed using the SPSS software program (version 23.0; SPSS, Inc., Chicago, IL, USA). 

## 3. Results

### 3.1. Characteristics of Native HDL and Glycated HDL

**Figure 1:**
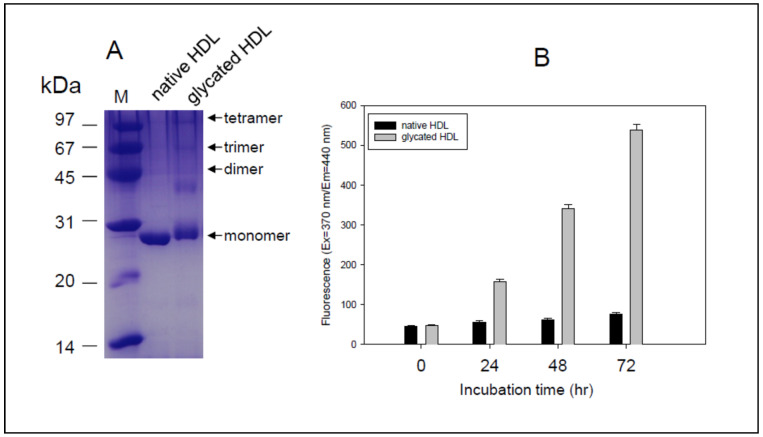
Characterization of HDL. (**A**) Electrophoretic patterns of native HDL and glycated HDL (12% SDS-PAGE). Lane M, molecular weight standard (Bio-Rad, low-range). (**B**) Fluorospectroscopic determination of glycation extent in HDL during 72 h (hrs).

After 72 h incubation in the presence of the fructose, glycated HDL showed a slight increase of molecular weight of apoA-I around 29–30 kDa with a smear band intensity. In contrast, native HDL showed 28 kDa of apoA-I with distinct and clear band intensity (Figure 1A). Glycated HDL also showed a multimerized band pattern up to tetrameric apoA-I, while native apoA-I showed a single band. Glycated HDL showed a rapid increase in yellowish fluorescence in a time-dependent manner during 72 h, which was approximately seven times higher than the native HDL (Figure 1B). These results indicate that the Maillard reaction had occurred in the HDL by the fructose treatment to produce multimerized apoA-I and advanced glycated end (AGE) product through the crosslinking of Lys and condensation of methylglyoxal, as reported previously [20,21].

### 3.2. Change HDL Particle and PON-1 Activity

**Figure 2:**
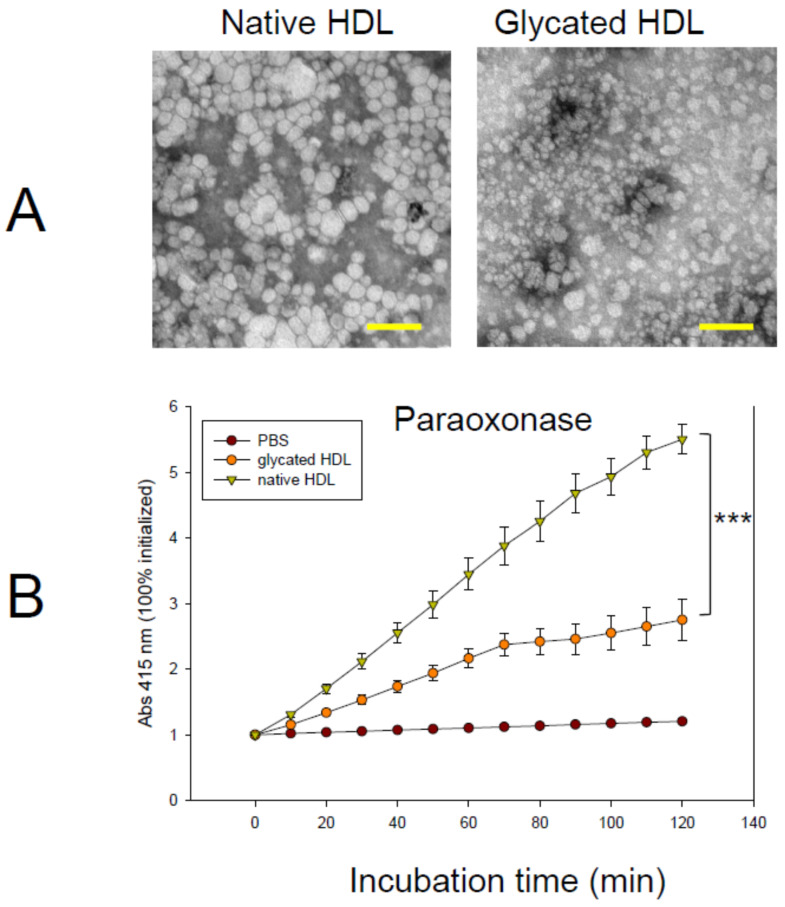
Observation of the HDL particle and antioxidant activity. (**A**) Negatively stained image of HDL from transmitted electron microscopy at a magnification of ×40,000. Scale bar (yellow) indicates 100 nm. (**B**) Changes in the activity of paraoxonase in HDL with or without glycation. The error bars indicate the SD from three independent experiments with duplicate samples. The molar extinction coefficient of p-nitrophenol was 17,000 M^−1^ cm^−1^. ***, *p* < 0.001.

The native HDL particle size was approximately 18–21 nm in length with a distinct particle shape (Figure 2A), while glycated HDL showed smaller particles, approximately 13–16 nm in length, with an ambiguous particle shape. This indicates that the glycation of HDL could induce a smaller particle size. In addition to particle size, the HDL-associated PON-1 activity was also decreased by glycation by up to 51% during 2 h incubation, as shown in Figure 2B. The PON-1 activity is associated with the antiviral activity against RNA viruses, such as HIV [30]. Similarly, patients with hemorrhagic fever renal syndrome (HFRS) showed a remarkable decrease in PON-1 activity in the oliguric phase up to 59–61% [6,7]. The current result shows a good agreement with previous reports to indicate that structural-functional correlations are critical to maintaining the PON-1 activity [31].

### 3.3. Uptake of acLDL 

**Figure 3:**
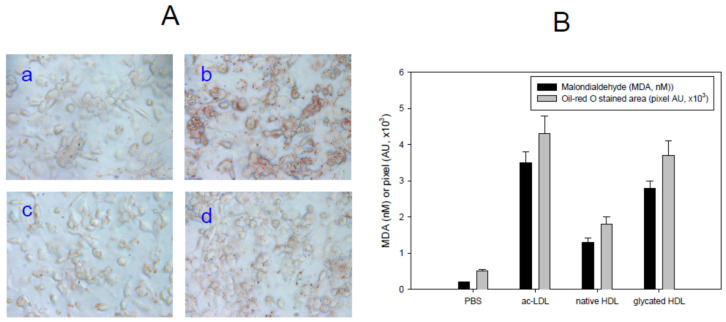
Anti-atherosclerotic activity with HDL. (**A**) Cellular uptake of acLDL in the presence of each HDL. Photo a, PBS alone; Photo b, acLDL-alone; Photo c, acLDL + native-HDL; Photo d, acLDL + glycated-HDL. (**B**) Determination of oxidized species in the cell media as a malondialdehyde standard. Oil-red O-stained cellular areas of 0.8 mm^2^ were quantified using computer-assisted morphometry. Data are shown as the mean ± SD of three independent experiments performed in triplicate.

As shown in Figure 3A, the ac-LDL-treated cells (Photo b) showed a much greater lipid-stained area with stronger red intensity than the PBS-treated cells (Photo a) as a control. In the presence of the same amount of acLDL, native HDL-treated cells showed a 60% smaller lipid-stained area (Photo c), suggesting that native HDL can inhibit the phagocytosis of LDL, which is the initial step of atherosclerosis. On the other hand, glycated HDL-treated cells showed 1.3 times more phagocytosis of LDL than the native HDL treatment. The production of oxidized species was highest in the acLDL alone treated group (photo b), approximately 3.8 nM of MDA. Under the same conditions, native HDL treated cells, or glycated HDL treated cells showed 1.3 nM or 2.8 nM of MDA in the cell media (Figure 3B). These results showed good agreement with previous studies showing that the glycation of HDL caused a loss of antioxidant and anti-atherosclerotic activity with more phagocytosis of LDL [20,21]. 

### 3.4. Vero E6 Cell Viability and Antiviral Activity 

**Figure 4:**
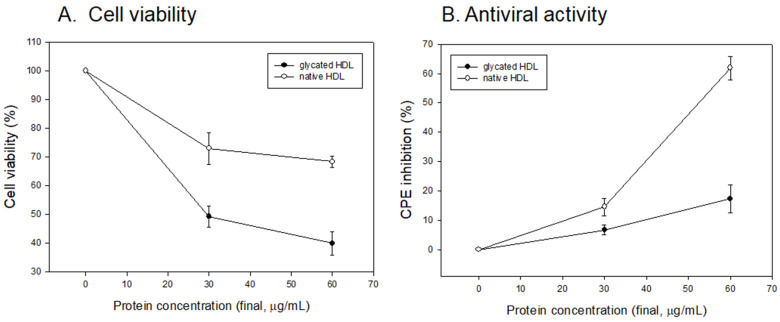
Comparison of glycated HDL and native HDL. (**A**) Cell viability of Vero E6 cell after treatment with lipoproteins. MTT cytotoxicity assays were carried out using uninfected Vero E6 cells with increasing concentrations of each lipoprotein. (**B**) Cytopathic effect (CPE) reduction activity of lipoproteins against SARS-CoV-2.

As shown in Figure 4A, the glycated HDL treatment showed the lowest cell viability around 49% and 40% under a final 30 and 60 μg/mL of protein, respectively, while native HDL showed 73% and 68%, respectively. This result suggests that more advanced glycated end (AGE) products, which are more cytotoxic, were produced in the glycated HDL. As well as the cytoprotective activity, native HDL showed potent antiviral activity against SARS-CoV-2 (Figure 4B) around 15% and 62% of CPE inhibition activity under a final 30 and 60 μg/mL of protein, respectively. However, glycated HDL showed remarkably decreased antiviral activity around 6% and 17% of CPE inhibition under a final 30 and 60 μg/mL of protein, respectively. 

### 3.5. Antiviral Activity against SARS-CoV-2 of Native HDL

**Figure 5:**
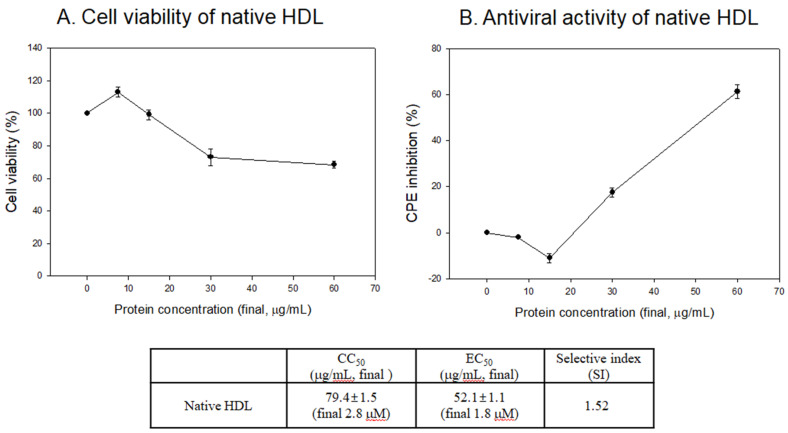
Vero E6 Cell viability (**A**) and cytopathic effect (CPE) reduction activity (**B**) of native HDL. EC_50_, effective concentration of compound needed to inhibit the CPE to 50% of control value. CC_50_, the cytotoxic concentration of the compound that reduced cell viability to 50%. SI, selectivity index = EC_50_/CC_50_.

As shown in Figure 5A, native HDL showed almost 100% cell viability under 7.5 and 15 μg/mL of protein. The cell viability was maintained 73% and 68% under 30 and 60 μg/mL of protein, respectively. Native HDL showed noticeable CPE inhibition ability of approximately 62% under 60 μg/mL treatment, which is 3.6-fold higher than that of glycated HDL. This result indicates that native HDL had potent antiviral activity with a selective index (SI) of native HDL of 1.52. On the other hand, it was impaired by AGE products via the glycation process.

Interestingly, the EC_50_ of native HDL, around 0.052 ± 0.001 mg/mL, was relatively lower than the physiological serum level of apoA-I around 1.04 mg/mL [32] and 1.3 mg/mL [30] from ordinary subjects as a control. This suggests that the serum concentrations of apoA-I/HDL-C are sufficient to display potent antiviral activity, provided HDL could maintain its native state with adequate PON-1 activity. Traditional CVD risk factors, such as smoking, diabetes, and hypertension, could help raise the morbidity and mortality from SARS-CoV-2 infections [33,34]. 

## 4. Discussion

Inflammation is a fundamental cause of almost all chronic metabolic disorders, and plays a major role in cardiovascular disease, cancer, rheumatoid arthritis, and chronic age-dependent disease [35]. Dyslipidemia and diabetes are risk factors of cardiovascular disease (CVD) by triggering an immune response [35]. HDL and apoA-I are strong antioxidant and anti-inflammatory molecules within the plasma [36]. On the other hand, systemic inflammation lowers the antioxidant and anti-inflammatory activity by transforming HDL to a pro-oxidant, pro-inflammatory acute-phase HDL [37], particularly in HIV-1 positive patients [19]. The antioxidant and anti-inflammatory activities of HDL are reduced significantly during the progression of influenza [38] and acquired immunodeficiency syndrome [39]. As the innate immunity, HDL-C inhibits toll-like receptor (TLR)-induced production of pro-inflammatory cytokines by macrophages [40]. In the same context, a lower HDL-C level in COVID-19 patients was correlated with a higher risk of a cytokine storm in the acute phase during a hepatic injury [41].

A few candidate drugs to treat COVID-19, such as chloroquine (CQ), hydroxychloroquine (HCQ), ivermectin, and baricitinib, commonly showed an elevation of serum HDL-C in human and animal models. CQ and HCQ have been used widely to treat infections, rheumatoid arthritis (RA), and systemic lupus. They increased LDL removal from plasma and the elevation of HDL-C in systemic lupus patients [42]. Ivermectin, an anti-parasitic drug, inhibited the replication of SARS-CoV-2 in vitro using Vero-hSLAM cells [43]. Ivermectin is a novel farnesoid X receptor (FXR) ligand that regulates the activation of the cholesterol metabolism [44]. Baricitinib, a TNF-α agonist and a drug for RA, also has been approved recently to treat COVID-19. Although the mechanism is still unclear, baricitinib could elevate the serum HDL-C in a dose-dependent manner [45]. These reports suggest that the antiviral activities of currently available FDA approved drugs are closely associated with an elevated serum HDL-C level.

In addition to the HDL-C quantity, enhanced HDL functionality with antioxidant activity, such as PON-1, is important to keep antiviral activity. On the other hand, HDL glycated by a methyglyoxal treatment showed a loss of PON activity [46]. Moreover, the lower PON activity was associated with a lower protective effect against oxidative damage and homocysteinylation. Interestingly, serum fructose concentrations in patients with diabetes were significantly higher than those in healthy subjects and non-diabetic patients [47]. HDL from elderly group showed smaller particle size and less PON-1 activity than those of young group [15]. HDL from elderly group showed truncated and multimerized apoA-I with higher glycation extent [16]. Similarly, Razavi et al. reported that PON-1 activity had negative correlations with HDL particle size, while serum HDL-C level was positively correlated with HDL particle size [48]. Taken together, those reports and current results indicate that HDL quality with PON-1 activity and particle size may be helpful for better understanding of COVID-19 risk and underlying disease, such as hypertension, diabetes, and coronary heart disease. 

Glycated HDL (Figure 1) showed a smaller particle size and loss of PON-1 activity (Figure 2) to display the atherogenic properties (Figure 3). The cytotoxic effects and loss of antiviral activity (Figure 4) of glycated HDL could be induced by NF-κB activation and cytokine production, such as TNF-α, IL-6, and ICAM, in macrophages in vitro, as reported previously [49]. Overall, those results suggest that the pro-inflammatory effects of modified HDL with a loss of PON activity were mediated by cellular signaling between the blocking of SR-BI and the binding of glycated HDL [50]. Future studies should be carried out to confirm antiviral activity of HDL with or without PON-1 activity. 

## 5. Conclusions

Native HDL with antioxidant and anti-atherosclerotic activity displayed potent antiviral activity to suppress the replication of SARS-Co-V2, while glycated HDL lost its antiviral activity. Therefore, it can be concluded that maintaining the structural-functional correlations of HDL with antioxidant activity may be critical for maximizing the antiviral activity against SARS-CoV-2.

## Data Availability

The data presented in this study are available within the article.
